# Influence of Pain, Risk Factors, and Functional Ability on Physical Activity Levels in Women with Anterior Knee Pain: A Cross-Sectional Study

**DOI:** 10.3390/medicina60091467

**Published:** 2024-09-07

**Authors:** Amjad Hajaj Alharbi, Mohamed K. Seyam, Ahmad Alanazi, Ahmed Almansour, Shahnaz Hasan

**Affiliations:** Department of Physical Therapy and Health Rehabilitation, College of Applied Medical Sciences, Majmaah University, Al Majmaah 11952, Saudi Arabia; 431204612@s.mu.edu.sa (A.H.A.); m.seyam@mu.edu.sa (M.K.S.); aalanazi@mu.edu.sa (A.A.); ah.almansour@mu.edu.sa (A.A.)

**Keywords:** anterior knee pain, activity level, risk factors, function ability, patellofemoral pain

## Abstract

*Background and Objectives*: Anterior knee pain (AKP) refers to chronic prepatellar pain and is one of the most common knee complaints in physically active women. This condition can significantly affect daily activities and overall quality of life. This study aims to assess the impact of pain, risk factors, and functional ability on different levels of physical activity (comparing low versus moderate activity) in women with AKP. *Materials and Methods*: This cross-sectional study involved fifty-six women diagnosed with AKP (aged 20–45 years) who were equally allocated into low and moderate physical activity groups. Their AKP and functional ability were assessed using the visual analog scale (VAS) and double squats and step-down tests, respectively. Possible risk factors were assessed using the Q-angle, modified Thomas test, sit and reach test, and patellar glide test. A person’s correlation coefficient and independent *t*-tests were used to determine the relationship and the differences between these variables while keeping the confidence interval level at 95%. *Result:* Women with moderate activity levels showed significantly higher scores on the VAS than those with low activity levels (*p* = 0.040). However, both groups had no significant difference in their functional ability or potential risk factors (*p* > 0.05). Additionally, their functional ability (double squat) showed a positive association with hamstring flexibility (Pearson correlation coefficient [r]:0.3; *p* = 0.006). *Conclusions*: Women with AKP who were engaged in moderate physical activity experienced higher levels of pain compared to those with low activity levels. These findings underscore the urgent need for further investigation into different levels of physical activity to develop appropriate prevention and treatment strategies for women with AKP.

## 1. Introduction

Anterior knee pain (AKP) accounts for 10–25% of all physical therapy clinic visits. It is a clinical syndrome known as peripatellar or retro-patellar pain, and is characterized by a dull, achy pain caused by changes in the physical and biomechanical components of the patellofemoral joints. The pain is often related to activity, begins gradually, and can be exacerbated by stair climbing and squatting [1,2,3]. AKP is often used interchangeably with patellofemoral pain syndrome (PFPS) [4]. Globally, the incidence of AKP is 22.7% in the general population and may worsen during adolescence, affecting 20–30% of people [4,5]. In Saudi Arabia, the rate of AKP is approximately 30.3% [6]. Primarily, it affects young individuals, commonly athletes, between the ages of 15 and 30 years, and women report this condition more frequently than men, at a ratio of 2:1 [7,8]. AKP is described as anterior pain in the knee (stabbing, retro-patellar or peripatellar, on-irradiative, and occasionally intermittent). It is exacerbated during weight-bearing tasks causing repetitive and high patellofemoral compressive forces [8,9,10]. Long-lasting pain persists for up to 16 years after the original diagnosis [11]. Several pathologies lead to AKP, such as patellofemoral tendon disease, runner’s knee, chondromalacia patellar, and patellofemoral pain syndrome, whether they are linked to pathologies like knee osteoarthritis or not [12,13]. AKP is a multifactorial syndrome, which means that several intrinsic and extrinsic risk factors influence it, such as movement-related factors, with activities involving a knee bend greater than 60 degrees and knee movement in front of the toes considered extrinsic risk factors [14]. Intrinsic risk factors are connected to a person’s physical and psychological characteristics. Moreover, a weak vastus medialis oblique, tight gastrocnemius–soleus muscle complex, dysfunctional hip muscles, foot pronation, generalized joint laxity, imbalanced limb length, and misaligned patella are all associated with this condition [15].

Psychological factors, such as depression and anxiety, may be indirectly related to AKP. However, physical activity levels and overuse injuries appear to stimulate the development and exacerbation of PFP [9]. This occurs mainly in activities that produce more substantial compressive pressures on the patellofemoral joint (PFJ), like those involved in some sports activities [16].

People diagnosed with AKP frequently endure crippling effects that may negatively impact their daily activities. Deficits in strength, range of motion, quality of movement, and postural control during functional activities are just a few of the many impairments reported in patients with AKP [17]. Moreover, it is reported that approximately 74% of AKP patients change or stop their activities due to knee pain [11].

Although physical activity levels may stimulate the exacerbation of AKP, there are limited data regarding the differences in pain, functional ability, and potential risk factors between low and moderate physical activity patients with AKP [18].

Recently, Saudi Arabian society has become more aware of the importance of sports and their tremendous role in better health and quality of life; various sports are practiced incorrectly, resulting in increased episodes of injuries. One of the most common symptoms seen recently in orthopedic physiotherapy clinics is AKP, which varies depending on the activity’s intensity and environmental setting [19]. AKP during adolescence can lead to decreased physical activity and impairment [20]. The major risk factors for AKP/PFPS are a malalignment of the lower extremity (patellar alignment, muscular imbalance, and over-activity), obesity, and hormonal imbalances. These risk factors predominantly affect individuals between 16 and 50 years old. This condition is increased in women aged 16 to 50 years [21]. There is limited research that has investigated the differences in pain and functional ability and potential risk factors for AKP between women with low and moderate levels of physical activity, and also limited studies that examine the relationship between risk factors, pain, and functional ability in response to the level of physical activity in women with AKP. This study hypothesizes that women with moderate levels of physical activity will experience lower levels of pain and have a better functional ability and different risk factors compared to women with low levels of physical activity. Additionally, it is hypothesized that there will be a positive correlation between physical activity levels and functional ability, and a negative correlation between physical activity levels and pain severity.

The primary purpose of this study was to evaluate the differences in pain, functional ability, and potential risk factors for AKP between women with low and moderate levels of physical activity. The second aim was to examine the relationship between these risk factors, pain levels, and functional ability in response to physical activity in women with AKP.

## 2. Methodology

### Study Design

This study used a cross-sectional design and was conducted at Qassim National Hospital and Alkheraif Center in Qassim City. Based on specific inclusion and exclusion criteria, 56 female participants with AKP were included. In this study, AKP is used as an umbrella term for all non-traumatic pain in the anterior knee area.

The study inclusion criteria were as follows: women with unilateral or bilateral AKP (one with more severe AKP); aged 20 to 45 years; displaying symptoms while performing at least two daily activities, such as during climbing and descending stairs, squatting, kneeling, jumping, and prolonged sitting [21]; an AKP duration lasting between four and eight weeks [16]; and who have passed integrity tests for their knee ligaments and other related structures of the knee complex. Women with pregnancy; a history of hip, knee, or ankle muscle, ligament, and meniscal injuries; contracture; rheumatoid arthritis; or neurological disorders; and those who were non-cooperative were excluded from the study.

The participants were categorized into moderate and low physical activity groups using the short self-administered Arabic version of the International Physical Activity Questionnaire.

The sample size was calculated using magnitude of effect size from a previous study [22]. For a large effect size (d = 0.8) at a power of 80% and an Alpha level (α) 0.05, a minimal sample size of 56 needed to be recruited [9].

The study complied with the Declaration of Helsinki guidelines and was approved by the Majmaah University for Research Ethics Committee under ethical approval number MUREC-jun8/COM-2023/21-4 dated 8/6/2023.

## 3. Instrumentation

Among many reliable and valid pain assessment tools, such as the numeric rating scale (NRS) or the numeric pain rating scale (NPRS), a visual analog scale (VAS) was used to assess the participants’ current AKP [1,23,24]. The tools used for the assessment include the visual analog scale (VAS), the Arabic version of the International Physical Activity Questionnaire (IPAQ-SF), an electronic goniometer, the sit and reach box test, a measuring tape, and the stopwatch app on a smartphone (iPhone 12Pro max).

## 4. Procedures

Data were collected for one lower extremity during one assessment session to minimize parameter testing variations. Before the physical examination, each participant diligently completed a comprehensive demographic questionnaire and self-reported tests of their pain and level of physical activity. The recorded information included the participants’ age, gender, weight, height, BMI, previous history of knee complex issues, the length of the current episode, and the location of their symptoms.

The intensity of their AKP was measured using the VAS, a self-reported measure recorded at one point along a 10 cm line. The left end (0 cm) represents “no pain”, and the right end (10 cm) represents “worst pain,” which is a reliable and valid scale for measuring pain [8,24,25].

The Arabic-translated version of the International Physical Activity Questionnaire (IPAQ-SF) is a general measure of physical activity and is recognized as a valid and reliable tool [26]. It consists of seven questions about the past seven days’ activities in domains such as physical activity related to jobs, transportation, housework, home maintenance, family care, sport and physical leisure activities, and time spent sitting. The total of the products between the expected energy expenditure and the number of hours spent on each activity (MET) provides an estimate of the kilocalories per kilogram used per day (kcal × kg^−1^ × d^−1^). The physical exercise dose is estimated in METs per minute per week (METs/min/week). The “last 7-day recall” version of the IPAQ-SF was suggested by its original authors for physical activity surveillance research because it places less burden on the participants to report their activities [16,27].

## 5. Physical Examination

To avoid test interactions, the order of the measurements performed during the physical examination were as follows: Q-angle, patellar glide test, Standard sit and reach test, modified Thomas test, step down, and double squat.

### 5.1. Q-Angle

We evaluated this with the subject supine and their knee completely extended. A universal goniometer was used to measure the angle in degrees that results from the intersection of their quadriceps force line (which runs from the anterior superior iliac spine to the center of the patella) and the patellar tendon center line (runs from the patella’s center to the tibial tubercle) [28]. The standard Q-angle should be generally between 12 and 20 degrees [29]. The validity and reliability of the clinical Q-angle is measured using the ICC range for inter-tester reliability, which is 0.20–0.70. The ICC range for intra-tester reliability is 0.22 to 0.75 [30].

### 5.2. Patellar Glide Test

The subject assumed a laying position, with their quadriceps relaxed and their knees extended. The therapist used their thumbs to apply a medially directed force to the patella’s lateral border, noting the most significant displacement of the patella’s inferior pole on the skin with a pen—afterward, a laterally directed force was applied to the patella’s medial border. The separation of these points was observed [31]. There are no recognized normative data for these metrics [32].

### 5.3. Standard Sit and Reach Test

This test is utilized to evaluate hamstring flexibility. Initially, participants were reclined with their legs fully extended and their soles pressed against the testing box. Then, participants were told to slowly reach as far forward as they could along the top of the box and maintain the position for two seconds. The participant’s score is determined by how fast their fingertips contact with the box. The maximum number was recorded after two trials in which the subjects’ knees were completely extended [33]. The average value ranged from 1 to 10 cm [34]. The sit and reach test is a valid tool for assessing hamstring flexibility [35].

### 5.4. Modified Thomas Test

This test is utilized to evaluate the quadriceps’ flexibility. Participants were ordered to sit at the edge of a therapeutic bed, turn over, and bring both knees to their chests. The test limb was lowered toward the floor while the unaffected limb was flexed entirely. The knee flexion angle was then evaluated using a goniometer, with the stationary arm aligned with the lateral midline of the thigh using the greater trochanter as a reference point. The lateral epicondyle of the femur serves as the fulcrum [33,36]. Hip flexor tightness at the two joints occurs when the knee cannot flex more than 80 degrees [37].

### 5.5. Step Down

This is a unilateral evaluation from an 8-inch height platform. Subjects step forward and down toward the floor. The lowered leg only makes light heel contact with the ground before fully extending the knee. This is a count of how many repetitions an individual completes in 30 s [38].

### 5.6. Double Squat

Subjects begin standing with their feet spaced shoulder-to-shoulder, knees fully extended, and their weight equally distributed on both limbs. Subjects lower their bodies to a knee position of ninety degrees and then return to full extension. In this test, every cycle from straight standing to ninety degrees of knee flexion and back to straight standing constitutes one cycle. We count how many bilateral lunges can be performed in 30 s [38].

### 5.7. Statistical Analysis

The data were analyzed using SPSS version 28.0. Descriptive statistics, including means, standard deviations (in case of the violation of normality), frequencies, and percentages, were calculated, and bar charts were utilized to display the data visually. The Shapiro–Wilk test was used to test the normality of the data. An independent samples *t*-test was employed to compare the differences between the moderate and low activity level groups. The Pearson correlation coefficient (r) was used to examine the association between pain, functional ability, and risk factors. Additionally, we compared the correlation among variables between the low and moderate physical activity groups when it comes to symptom locations, such as prepatellar, retro-patellar, and around the knee. Statistical significance was determined at a *p*-value < 0.05.

## 6. Results

**Figure 1:**
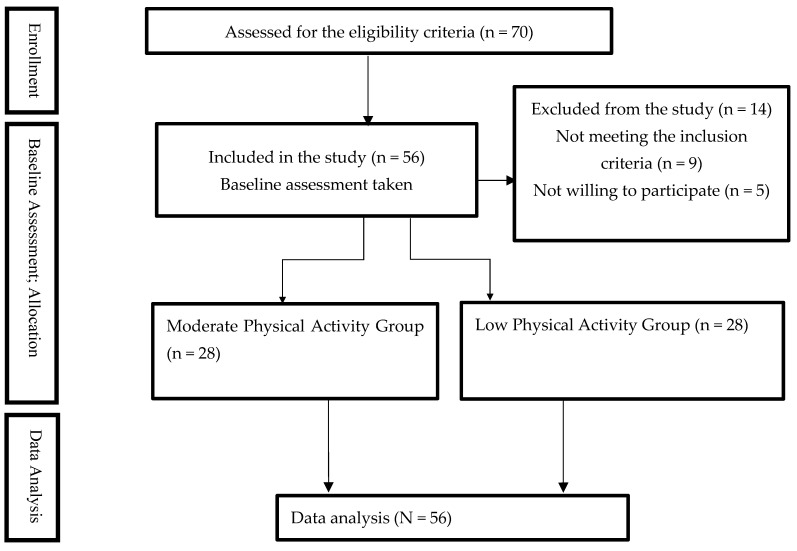
STROBE flow chart of study procedures, including participants’ enrollment, baseline assessment, and data analysis.

Seventy participants were screened for eligibility, and only fifty-six participants met the inclusion criteria and were equally allocated to the moderate physical activity group (n = 28) or low physical activity group (n = 28). The study procedures, including the participants’ enrollment, baseline assessment, and data analysis are presented in Figure 1.

The participants’ socio-demographic variables are shown in Table 1. This table compares the descriptive characteristics of the participants divided into two groups: the moderate physical activity group (n = 28) and low physical activity group (n = 28). This table presents mean and standard deviations (SDs) for continuous variables, and numbers (N) and percentages (%) for categorical variables. The *p*-value indicates the statistical difference between the two groups.

The mean age of the moderate activity group was 31.75 ± 6.53 years and that of the low physical activity group was 30.29 ± 5.02, while the mean BMI was 24.21 ± 3.82 in the moderate physical activity group and 25.1 ± 3.20 in the low physical activity group. The mean height of the moderate activity group was 158.1 ± 4.91 cm, while it was 158.7 ± 6.31 cm in the low activity group. Also, the mean weight of the moderate activity group was 61.75 ± 9.88, and this was 65.5 kg ± 8.80 in the low activity group. The duration of their symptoms was 31.7 ± 6.53 weeks in the moderate activity group and 30.29 weeks ± 5.02 in the low activity group.

**Figure 2:**
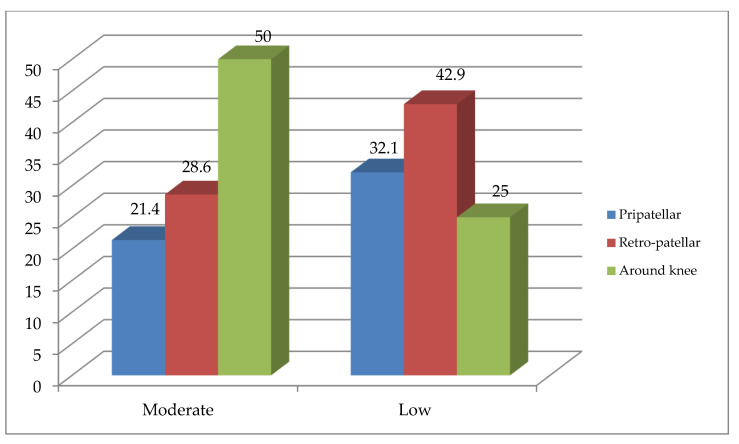
The location of symptoms in the moderate and low physical activity groups.

In addition, moderate physical activity group had 6 (21.4%), 8 (28.6%), and 14 (50%), whereas the low activity group had 9 (32.1%), 12 (42.9%), and 7 (25%), peripatellar, retro-patellar, and around-knee symptom locations, as shown in Figure 2 respectively. Finally, the mean physical activity level of the moderate physical activity group was 685.96 MET, while this was 505.57 MET in the low physical activity group.

### Between-Group Comparison (Moderate vs. Low Physical Activity)

An independent sample *t*-test revealed a significant difference (95% CI; *p* < 0.05) in the outcomes of the VAS between the moderate and low physical activity groups (t = 1.97; *p* = 0.040). However, it revealed insignificant differences (95% CI; *p* > 0.05) for the outcome scores of the functional ability tests, such as the double squat (t = 0.27; *p* = 0.783) and step down tests (t = 1.32; *p* = 0.191), and risk factors, such as the Q-angle (t = 1.10; *p* = 0.305), modified Thomas test (t = 1.78; *p* = 0.081), sit and reach test (t = 0.76; *p* = 0.446), and glide test (t = 0.03; *p* = 0.973), when comparing between the moderate and low physical activity groups, as demonstrated in Table 2.

According to Table 3, there was a significant weak positive association between functional ability (double squat) and a risk factor (sit and reach test) (r = 0.362; *p* = 0.006) and a significant weak negative correlation between functional ability (step down) and pain (r = −0.347; *p* = 0.009). No significant association was detected between functional ability (double squat), the Q-angle, and other risk factors (modified Thomas test and glide test). However, no significant association was found between pain and step down with respect to the risk factors (*p* > 0.05).

There are significant variations in the correlation patterns between the groups with different levels of physical activity (moderate vs low), as presented in Table 4. In the moderate physical activity group, there are strong negative correlations between BMI, physical activity level, and Q-angle when it comes to prepatellar and retro-patellar pain. Similarly, in the low physical activity group, there is a strong positive correlation between BMI and VAS and a moderate negative correlation between BMI and Q-angle when it comes to prepatellar, retro-patellar, and around-knee pain. The correlation between the participants’ physical activity level and Q-angle is mainly positive, indicating that moderate physical activity levels generally correspond to higher Q-angles. In the low physical activity group, there is a significant positive correlation between VAS, Q-angle, and around-knee pain, suggesting a strong relationship between pain intensity and the Q-angle.

## 7. Discussion

The purpose of this study was to evaluate the differences in the outcome scores, such as pain, functional ability (double squat and step down), and risk factors (Q-angle, Thomas modified test, sit and reach test, and glide test), between the participants from the low and moderate physical activity groups. The current study’s results demonstrated a significant difference in the VAS outcome scores between the participants with moderate physical activity and those with low physical activity. However, there was no statistically significant difference between the moderate and low physical activity groups’ outcomes in terms of their functional ability or risk factors.

Different levels of physical activity and its overuse have been discussed in some studies. Overloading and repetitive physical activity mainly trigger the occurrence and exacerbation of AKP [21]. In another study, reported that people suffering from prepatellar pain develop symptoms due to increased physical activity or loading on their knees for long periods [39]. Furthermore, Fairbank et al., 1984, reported that people with AKP reported that their maximum level of pain was associated with increased physical activity [40].

### 7.1. Physical Activity and Level of Pain

Regarding pain level, the results of the current study demonstrated that participants with AKP who maintain moderate levels of physical activity have higher levels of pain than those who maintain low physical activity levels. By reviewing the literature, we found that the exact cause of pain in patients with AKPS is unclear and not correlated specifically to any leading cause. A previous study reported substance P-rich free nerve endings within Hoffa’s fad pad, retinacula, and subchondral bone [41]. In another study, reported increased levels of neuronal markers such as neurofilament protein, nerve growth factor, and substance P in the lateral retinaculum of people with patellofemoral mal tracking [42]. This study shows that the development of AKP may be due to retinal innervation. One of a previous study hypothesized that mechanical stress within the retina is related to the change in the level of substance P [43]. However, other evidence confirms the role of subchondral bone in the increase in pain in patients with PFPS [44].

A prolonged duration of biomechanical alteration in the knee joint complex can result in chronic pain characterized by pain sensitization mechanisms, which can increase the perception of pain regardless of the level of physical activity. In the moderate physical activity group, the longer duration of their symptoms may increase the pain felt due to factors such as central sensitization, tissue damage, and maladaptive pain coping mechanisms. Furthermore, persistent symptoms can lead to reduced physical function and increased disability, further impacting the severity of their pain. Therefore, the duration of symptoms may partly explain the higher pain levels observed in the moderate physical activity group, potentially complicating the relationship between physical activity and pain [45].

The location of pain and secondary hyperalgesia can significantly influence pain severity in women with anterior knee pain, particularly in the context of moderate physical activity. Anterior knee pain often affects the patellofemoral joint, where localized pain can be exacerbated by repetitive stress from physical activities. The presence of secondary hyperalgesia, characterized by an increased sensitivity to pain in areas surrounding the initial injury site, may amplify the overall experience of pain. This heightened sensitivity can result from neural sensitization, where the central nervous system becomes more responsive to pain stimuli, leading to a broader and more intense pain experience. In women with anterior knee pain, this combination of localized pain and secondary hyperalgesia may contribute to greater discomfort during activities that involve knee loading, such as walking or climbing stairs, thereby affecting both the severity of their pain and their functional mobility. Understanding these factors is crucial for creating effective pain management and rehabilitation strategies [46].

Based on previous studies, we can explain the current results concerning the increased pain level in moderate physical activity participants compared to that in low physical activity level participants due to the high susceptibility of the PFJ to being overloaded during moderately physical activities. This explanation was confirmed that articular cartilage deterioration and many knee joint diseases, such as PFP, may be associated with excessive joint stress [47]. Our results are in agreement with a previous study reported that adolescents with AKP had higher levels of pain associated with increased physical activity [40]. Furthermore, our results were supported by another study, reported that intense physical activity appears to be more associated with knee pain than moderate physical activity [9]. In addition, the current study’s results align with a recent study, who found that adolescent athletes presented higher pain levels than physically active non-athletes [16].

### 7.2. Physical Activity and Functional Abilities

It has been hypothesized that pain has an impact on the physical activity levels of people with AKP, and it has been found that between 71 and 74% of those people change or even stop their physical activity [48]. However, in the current study, there was no significant difference in functional ability between participants with moderate physical activity levels and those with low activity levels. This can be explained depending on the cause of pain in the low activity level group, which may be due to psychosocial factors, such as a fear of increasing their pain with increased activity or during normal daily activities [28].

In the current study, we evaluated the participants’ functional abilities using two functional performance tests (the step down and bilateral squat), which are not real-life normal functional activities such as walking, sitting for long periods, and going up or down stairs. Additionally, these tests are subjective, not objective, which is one study limitation. A study was designed to determine the reliability of functional performance tests for people with AKPS, they found that the bilateral squat test was not significantly related to their level of pain, which means that this test was not sensitive to any changes that may occur in the participant’s level of pain [38]. Based on their results, we can interpret why, even though the pain significantly differed between both groups, there were no significant differences in the groups’ functional abilities.

### 7.3. Physical Activity and Anterior Knee Pain Risk Factors

Many factors can cause AKPS, including muscle flexibility, structural abnormalities, and kinematic variables [13,49]. Regarding the reported risk factors in the current study (Q-angle, muscle flexibility, and patellar gliding), there was no significant difference between participants with moderate physical activity and those with low activity levels in any of the measured risk factors. Furthermore, regarding the association between pain, functional ability and the reported risk factors, there was only a significant weak positive association between function ability (double squat) and hamstring flexibility (the sit and reach test) (*p* = 0.006). In contrast, no significant association between function ability (double squat and step down) or any other risk factor was detected. Also, no significant association was found between pain and all reported risk factors (*p* > 0.05). The lack of a difference between the two groups in terms of the measured risk factors and the lack of a significant association between them and pain and functional ability in the current study can be explained by relying on the contradictions observed between the results of studies that discussed the risk factors in cases of AKP in terms of different individual characteristics and their association with their physical performance [50,51].

The Q-angle is considered one of the most clinically reliable standards in determining the level of strength of the quadriceps muscle and is one of the most critical factors affecting the patellofemoral joint. In addition, it is considered an indicator of performance in athletes and used in diagnosing many painful disorders and diseases of the PFJ. However, there are contradictions in the results of studies regarding its effect and variation between subjects with AKPS [52,53]. In a study, the authors found a statistically significant increase in the Q-angle in subjects with PFPS compared to subjects in the control group [52]. However, another study, found no significant difference between groups, which is in agreement with the results of the current study [53].

Furthermore, our results were supported by a study, as they reported no significant difference in the magnitudes of the Q-angle between participants with future AKPS and controls [54]. Additionally, some prospective studies have examined the effect of the Q-angle on the incidence of PFPS in active populations, monitored 282 physical education students over two years. They found that only 24 students developed AKPS. They concluded that lower leg alignment, including the Q-angle, is not associated with the development of AKPS, which aligns with our results [38,55].

On the other hand, the quadriceps muscle is considered one of the most important soft tissue structures that has a significant role in the normal functioning of the PFJ due to its ability to pull the patella in the superior and lateral directions [56,57]. So, quadriceps tightness may cause high patellofemoral stresses, predisposing individuals to developing symptoms [58]. In a study, researchers stated that decreased quadriceps muscle flexibility was present before the onset of symptomatic syndromes and, therefore, not necessarily a consequence of AKPS [55]. The presence of quadriceps tightness as a risk factor for developing AKPS was also a contradiction between studies. It has been reported in a previous study that quadriceps tightness is considered a risk factor for developing AKPS in adolescent elite figure skaters, while another study reported no significant difference in quadriceps tightness between participants [59,60].

Another risk factor is hamstring tightness, which creates a greater passive resistance in the hamstring that has been proposed to either require greater quadriceps efforts to overcome or a minor bending of the knee during exercises, both of which may increase the reaction forces on the PF joint [61]. Our results regarding hamstring tightness align with a previous study report that found hamstring tightness was not a significant risk factor for developing AKP in an athletic population. At the same time, our results contradict with another study that reported hamstring tightness has a significant effect as a risk factor for AKP/PFPS athletes [55,61].

Regarding patellar glide/mobility, which was the last risk factor reported in the current study, we reported no significant difference between participants with moderate physical activity and those with low physical activity levels in the patellar gliding test. Our results are supported by a previous study, reported that although the medial, lateral, and total patellar mobility were greater in participants with PFPS, these findings were insignificant [55].

## 8. Limitations of the Study

The first limitation of the current study is that it was conducted on females only. Therefore, we cannot generalize our results, as it has been reported that there is a gender effect on the incidence and prevalence of AKP [62]. The findings also were limited in determining the functional abilities of the participants as they relied on only two tests of their functional abilities, which are not actual functional activities of daily life, such as walking, going up or down the stairs, and sitting for long periods, which could have a more significant impact on determining the difference in the effect of the level of activity among participants. Another limitation is the lack of accuracy in determining the actual activities of the participants, who classified their activities as “moderate” and “low” and evaluated them in general without specifying actual activities and studying the extent of their association with the occurrence of their AKP. Additionally, the exclusion of psychological factors was a limitation, as these factors are known to potentially influence the experience of pain and functional outcomes in women with AKP.

### The Implementation of Treatment

Based on the findings of the current study, there are some relevant points that researchers and clinicians should take into consideration. First, when conducting studies on females who suffer from AKP, their physical activity levels should be assessed. In addition, when they are distributed into groups during the study, they should be grouped by the same level of physical activity, particularly when their pain level is assessed. Second, if the study sample consists of women with AKP who perform any degree of physical activity, a PFJ loading protocol must be used to avoid any difference in pain level between the participants.

## 9. Conclusions

Overall, the data suggest that BMI significantly influences physical activity levels and Q-angle when it comes to pre-patellar AKP; it also influences the Q-angle when it comes to retro-patellar AKP in women with moderate physical activity. Similarly, BMI influences the VAS and Q-angle when it comes to pre-patellar and retro-patellar AKP in the low-activity group. Moreover, the Q-angle influences the VAS when it comes to AKP around the knee in women with low activity.

These findings suggest the need for further investigation into different levels of physical activity to develop appropriate prevention and treatment strategies for women with AKP. Their physical activity levels should be assessed by incorporating the use of wearable devices to track their daily step counts. This approach would provide a more accurate and continuous measure of their physical activity levels, allowing for better correlation with their pain levels, functional ability, and other study outcomes. Psychological factors must be included in the assessment to provide a more comprehensive understanding of the study’s scope and the potential factors influencing the results.

## Figures and Tables

**Table 1:** Participants’ demographic characteristics.

Characteristics	Moderate (n = 28)		Low (n = 28)	*p*-Value (Independent Sample *t*-Test)	
	Mean	SD	Mean	SD	
Age (years)	31.75	6.53	30.29	5.02	0.351
Height (cm)	158.71	6.31	158.18	4.91	0.724
Weight (kg)	65.54	8.80	61.75	9.88	0.136
BMI (kg/m^2^)	25.11	3.20	24.21	3.82	0.348
Duration of symptoms (week)	31.75	6.53	30.29	5.02	0.033 *
Physical activity level(MET∙min∙wk^−1^)	685.96	51.75	505.57	43.64	<0.001 *
Previous injury	Number (%)			0.284 (Chi-square test)	
Yes	13 (46.4%)		17 (60.7%)		
No	15 (53.6%)		11 (39.3%)		
Location of symptoms	Number (%)			0.155(Chi-square test)	
Prepatellar	6 (21.4%)		9 (32.1%)		
Retro-patellar	8 (28.6%)		12 (42.9%)		
Around knee	14 (50%)		7 (25.0%)		

*—Significant value, if *p* < 0.05.

**Table 2:** Difference in VAS, functional ability, and risk factors according to participants’ level of activity.

Level of Activity	Low	Moderate	T-Value	*p*-Value (Independent Sample *t*-Test)
	Mean ± SD	Mean ± SD		
VAS	5.20 ± 1.81	6.00 ± 1.19	1.97	0.040 *
Double squat	12.04 ± 3.9	11.75 ± 3.83	0.27	0.783
Step down	11.54 ± 3.33	10.00 ± 5.16	1.32	0.191
Q-angle	12.57 ± 3.73	13.54 ± 3.23	1.1	0.305
Modified Thomas test	64.79 ± 6.71	60.75 ± 9.9	1.7	0.081
Sit and reach test	23.73 ± 5.26	25.07 ± 7.58	0.76	0.446
Glide test	1.45 ± 0.34	1.45 ± 0.44	0.03	0.973

*—Significant value, if *p* < 0.05.

**Table 3:** Association between pain, Q-angle, functional ability, and risk factors.

Variables	Correlation with Pain and Functional Ability					
	Pain		Double Squat		Step Down	
	r-Coefficient	*p*-Value	r-Coefficient	*p*-Value	r-Coefficient	*p*-Value
Q-angle	0.253	0.60	−0.113	0.409	0.039	0.775
Modified Thomas Test	−0.172	0.206	−0.100	0.464	−0.167	0.219
Sit and Reach Test	−0.026	0.850	0.362	0.006 *	0.179	0.186
Glide Test	0.052	0.706	0.232	0.085	0.005	0.973
Pain	1	-	−0.045	0.741	−0.347	0.009 *

*—Significant value, if *p* < 0.05.

**Table 4:** Correlation comparison of symptom locations (1 = prepatellar, 2 = retro-patellar, 3 = around-knee) with different variables (BMI, PA level, Q-angle, and VAS) between moderate PA and low PA groups (N = 28/group; 95% Cl).

Correlation	PA-Mod-1	PA-Mod-2	PA-Mod-3	PA-Low-1	PA-Low-2	PA-Low-3
BMI vs. PA level	−0.912	−0.003	0.136	−0.215	−0.145	−0.095
BMI vs. VAS	0.291	0.311	0.156	0.650	−0.198	−0.392
BMI vs. Q-angle	−0.879	−0.600	0.421	−0.642	−0.252	−0.321
PA Level vs. VAS	−0.379	−0.106	0.496	−0.428	0.262	−0.026
PA Level vs. Q-angle	0.688	−0.248	0.181	0.178	0.166	0.049
VAS vs. Q-angle	−0.088	0.336	0.080	−0.369	0.458	0.616

## Data Availability

The dataset that was analyzed or the results presented this study are available upon a reasonable request from the corresponding author.
